# Clinical Holistic Medicine: The Case Story of Anna. I. Long-Term Effect of Childhood Sexual Abuse and Incest with a Treatment Approach

**DOI:** 10.1100/tsw.2006.329

**Published:** 2006-02-02

**Authors:** Søren Ventegodt, Birgitte Clausen, Joav Merrick

**Affiliations:** ^1^ The Quality of Life Research Center, Teglgårdstræde 4-8, Copenhagen K, Denmark; ^2^ Research Clinic for Holistic Medicine and Nordic School of Holistic Medicine, Copenhagen, Denmark; ^3^ The Scandinavian Foundation for Holistic Medicine, Sandvika, Norway; ^4^ Vejlby Lokalcenter, Vejlby, Denmark; ^5^ National Institute of Child Health and Human Development, Faculty of Health Sciences, Ben Gurion University, Beer-Sheva, Israel; ^6^ Center for Multidisciplinary Research in Aging, Faculty of Health Sciences, Ben Gurion University, Beer-Sheva, Israel; ^7^ Office of the Medical Director, Division for Mental Retardation, Ministry of Social Affairs, Jerusalem, Israel

**Keywords:** quality of life, QOL, philosophy, human development, holistic medicine, public health, holistic health, holistic process theory, life mission theory, incest, sexual abuse, rape, borderline, induction of recovery, group therapy, Denmark

## Abstract

The nervous breakdown of a 22-year-old, young woman was caused by severe sexual abuse in childhood, which was repressed over many years. During therapy, the patient accumulated resources to start the painful integration of these old traumas. Using holistic existential therapy in accordance with the life mission theory and the holistic process theory of healing, she finally was able to confront her old traumas and heal her existence. She seemingly recovered completely (including regaining full emotional range) through holistic existential therapy, individually and in a group. The therapy took 18 months and more than 100 hours of intensive therapy. In the beginning of the therapy, the issues were her physical and mental health; in the middle of the therapy, the central issue was her purpose of life and her love life; and at the conclusion of the therapy, the issue was gender and sexuality. The strategy was to build up her strength for several months, mobilizing hidden resources and motivation for living, before the old traumas could be confronted and integrated. The therapy was based on quality of life philosophy, on the life mission theory, the theory of ego, the theory of talent, the theory of the evil side of man, the theory of human character, and the holistic process theory of healing. The clinical procedures included conversation, philosophical training, group therapeutic tools, extended use of therapeutic touch, holistic pelvic examination, and acceptance through touch was used to integrate the early traumas bound to the pelvis and scar tissue in the sexual organs. She was processed according to 10 levels of the advanced toolbox for holistic medicine and the general plan for clinical holistic psychiatry. The emotional steps she went through are well described by the scale of existential responsibility. The case story of Anna is an example of how even the most severely ill patient can recover fully with the support of holistic medical treatment, making her feel, understand, and let go of her negative beliefs and life-denying decisions.

## INTRODUCTION

The scientific breakthrough in the understanding of human sexuality, still a most central theme in the clinic of the general practitioner[1], came with the brilliant work of Masters and Johnson in the middle of the last century[2,3]. The most famous curve in sexological research is still the curve of the male and female sexual reaction cycles, explaining the four phases of the normal sexual intercourse: the excitement phase, the plateau phase, the orgasmic phase, and the relaxation phase. Since their work, most clinical sexologists have recognized a prephase of lust, where one of the most dominant problems of our time is the lack of sexual lust in the female[4]. We have recently presented a theory of sexuality that can serve as guidance for holistic sexologic therapy[5], especially when we want to treat the whole person and overview all the relevant dimensions of sexuality and existence. We want our patient to be a whole, balanced, ethical, and able person, not just to be able to function sexually. In our work with sexually abused patients, we have often found that severe mental disturbances and even insanity can follow directly from sexual abuse.

As sexual and existential problems often go hand in hand, and as both existence and sexuality are theoretically difficult issues, maybe the two most fundamental questions of the research in human life and quality of life are “What is existence?” and “What is sexuality?” Often the first question is left unanswered and the second met with theoretical answers from evolutionary theory and psychosocial models[6,7], which is very difficult to use in the holistic sexological clinic. The following case story shows how a combination of holistic sexology, holistic psychiatry, and advanced holistic existential therapy can be used to treat even the most severe cases of sexual abuse. We use the strategy for spontaneous regression in the holistic healing of patients with incest and rape traumas[8]. The patient was treated in accordance with the 10 steps of the advanced holistic toolbox[9].

Anna was a student, aged 22 years, who had completely repressed over 100 episodes of sexual abuse, incest, and rape throughout her early childhood[10]. She now seems to have recovered completely (including regaining her full emotional range) through holistic existential therapy, individually and in a group. The therapy took 18 months and more than 100 hours of intensive therapy. In the beginning of the therapy, the issues were her physical and mental health; in the middle of the therapy, the central issue was about her purpose of life and her love life. At the end of the therapy, the issue was gender and sexuality. The strategy was to build up her strength for several months, mobilizing all her hidden resources and motivation for living, before the painful old traumas were confronted and integrated. The therapy was philosophically based on quality of life philosophy[11,12,13,14,15,16,17,18] and theoretically based on the life mission theory[19], the theory of ego[20], the theory of talent[21], the theory of the evil side of man[22], the theory of human character[23], and the holistic process theory of healing[24,25,26], all of which she became familiar with during the therapy. The clinical procedures included conversation and philosophical training and the group therapeutic tools. Extended use of therapeutic touch, holistic pelvic examination[27], and acceptance through touch[28] were used to integrate the early traumas bound to the pelvis and scar tissue in the sexual organs. She was processed according to the general plan for clinical holistic psychiatry[29,30] and the steps she went through are described by the scale of existential responsibility (the Responsibility-for-Life Scale)[30].

The four fundamental steps of healing her were (1) loving her (or in other words intense care for the patient), (2) winning her trust, (3) getting permission to give her holding and support, and (4) reparenting, allowing her to be a child again, to feel, understand, and let go of literally hundreds of negative, life-denying decisions[19,31,32]. Her case is a fine example of the power of healing with holistic existential therapy. It describes the dangers and problems of working with love and intimacy with the sexually abused patient, but also the huge value of holistic gynecology and sexology in this situation[4,28,33].

## CLINICAL HOLISTIC MEDICINE

The life mission theory[19,20,21,22,23,34,35] states that everybody has a purpose of life or huge talent. Happiness comes from living this purpose and succeeding in expressing the core talent in life. To do this, it is important to develop as a person into what is known as the natural condition, a condition where the person knows himself and use all his efforts to achieve what is most important for him. The holistic process theory of healing[24,25,36,37] and the related quality of life theories[38,39,40] state that the return to the natural state of being is possible whenever the person gets the resources needed for existential healing. The resources needed are “holding” in the dimensions of awareness, respect, care, acknowledgment, and acceptance with support and processing in the dimensions of feeling, understanding, and letting go of negative attitudes and beliefs. The preconditions for the holistic healing to take place are trust and the intention for the healing to take place. Existential healing is not a local healing of any tissue, but a healing of the wholeness of the person, making him much more resourceful, loving, and knowledgeable of himself, his own needs, and his wishes. In letting go of negative attitudes and beliefs, the person returns to a more responsible existential position and an improved quality of life. The philosophical change of the person healing is often a change towards preferring difficult problems and challenges instead of avoiding difficulties in life[11,12,13,14,15,16,17,18]. The person who becomes happier and more resourceful often also becomes more healthy, more talented, and more able to function mentally, emotionally, physically, sexually, and spiritually[26,41,42].

## THE STORY OF ANNA

### The Struggle of an Abused Young Girl for Love and Sanity

Sometimes, there is a particular chemistry between the patient and the therapist that enables the patient to get released from even very serious circumstances. The story we are going to tell is one of the most intense and dreadful ones we have yet experienced in the Research Clinic for Holistic Medicine and, at the same time, the most beautiful and accomplished treatment we have given.

There were three major difficulties in the course of the therapy. In the beginning, it was extremely difficult to win the trust of this patient and only after months of therapy was this accomplished. Then, since her resources were so small and only after she was supported by a whole group of people (the group level[26] or the “level 5 of holistic medical tools”[9]) could she confront the real cause of her mental and emotional problems, namely, the repression of years of brutal sexual abuse in her childhood by three different men when she was 2–7 years old. When trust and intimacy finally were established in the group setting, the patient regressed so intensively into old painful wounds of sexual abuse that she almost lost her mind and entered into a psychotic paranoiac state, which took intensive resources over a month to heal with extensive use of the strategies for healing mental disease with holistic therapy[29,30]. At the end of the therapy, separation between patient and physician was emotionally difficult and, in the end, she felt somewhat rejected. To help this extraordinarily wounded patient, most of the more complex concepts of holistic medicine, including holistic rehabilitation, holistic sexology[4,28], and holistic psychiatry were used. Surprisingly, there were no traumas, even the most brutal, that could not be healed, seemingly completely, in the holistic existential therapy.

## Physician Rapport

The patient was a beautiful, slightly chubby, 22-year-old woman of average height, that we will call Anna. When she came to consult our clinic the first time, she was a student at the School of Education. She had started to get uncontrollable weeping outbursts for which she could not give any reason herself. Furthermore, she suffered from a low back condition, possibly a slipped disc of earlier date, which caused her much pain. She had also sexual problems. It was as if her life was going to overturn mentally, her zest for life diminished more and more and had nearly disappeared. As her confidence in other people drastically diminished, her contact to the outside world became more and more moderate and her thoughts got ever more strange. Most of all, it seemed to be early schizophrenia. It is not easy to say what her illness would have developed into if she had not been treated. Her condition was not steady and was worsening fast.

At the close of treatment, I (SV) could conclude her to be a happy, healthy, and natural young woman with renewed appetite for life and love. In this connection, I received permission to use her story for publication, as well as her own description of how she had experienced the treatment, also for this use and she gave me both[10]. Her case report is the purpose of this paper taken from the medical chart constructed during the treatment, while her own description of the treatment according to her diary will be in another paper.

## Medical Chart

**Female, 22 years old, suspected borderline/schizophrenic after physical maltreatment, incest and multiple rapes in early childhood:** First quality of life (QOL) conversation: Student at a School of Education. Prior history of slipped disc, of stable type and not in need of an operation. Has been painless for some years, now again problems with the back. Skin eruption on neck and chest of nettle-rash type. Come because she is very sad with uncontrollable weeping episodes. Feels as if she weighs 3,000 tons and now it has to come out, she cannot hold it back any longer. Hard for her to show me confidence, but it gets better along the conversation. Would like to attend a process here and I offer to work at her feelings as well. I presume that the low back/slipped-disc problems, which are probably due to tightening in the back, can be solved. New appointment in one week. Objective examination: Very hesitating, very sceptic, very “sweet” and self-sacrificing. Holistic medicine is explained. At the end of the consultation she allows me to touch her back where it hurts. She does not allow me to touch her stomach during the physical examination. EXERCISE: Life story. Write the episodes you remember, where you had emotional problems. What happened, what did you feel, what did you possibly conclude? New appointment in one week. EXERCISE: Keep private all what we talked about here.

Second conversation: Tightening in the back for which physiotherapy does not help. Has written her life story, which we discuss. Topic: lack of care and outrage, violent by father, as well as dysfunctional family. Cries and is glad for the intimacy for which I also thank her that she is opening up. On the plank bed we work at the feelings in the shoulders, breast and stomach. She allows me to get deeply. Talk about the centres and our resources. EXERCISE: Find your problematic life topics - trust/distrust, infringement/escape. Sexuality: control. EXERCISE: Write incidents with emotional contents; circumstances that are relevant for the topics.

Third conversation: Process: "I am not important". Her pattern was that only her father was important, and she used a huge amount of energy to look where her father was. Since last she has had some new sensations as if something has healed. Had brought an action against a colleague about sexual harassment, which she won. It was brought up by the managers. We talk about sex, she is not dating her fiancé any more and has no desire. When with him she did not think of herself or her own needs. EXERCISE: Do not compromise with yourself on sexuality; do only consent if you really want to. EXERCISE: Write about all the sexual defeats and see how they are actually connected with "I am not important".

4th conversation: Has been in touch with the psychiatric system where she received a tranquillizer. She had a very violent weeping episode, could not stop crying and her fiancé was worried. Laid crouching on the kitchen floor and could not stop weeping. We talk about containing the feelings and to make her fiancé to contain them as well. May call me on my mobile hot-line number if new troubles arise. May come weekly. EXERCISE: Works at containing the feelings. The feelings are OK. It is OK to feel sad, afraid etc. - and the feelings always stop again if only you get care, respect and give yourself the opportunity to sense.

5th conversation: Has had a hard time for six days. Stayed at home from school, but I ask her to go there again, be sensitive and strong in the feelings. If people want to judge her weak because she is sensitive, it is their problem. On the phone, she put her father in his place. Told him a few home truths and it has been really nice. Sex: She is doing a little better, but not good at just being there and enjoying it - and expressing lust and pain through sounds. She is used to perform sex. Conversation about just being present and letting him enjoy you when he wants and in the way he wants - and expressing no and yes sincerely and continuously. EXERCISE: Express no and yes when having sex, generally say to and fro.

6th conversation: Arrive in a poor state, weeps and is sad. Tells me she is such a softie and has so little to give to others. However, she seems to be much more attentive, she fills up the space better now, so in my opinion she is making progress. She is doing fine with the no and yes exercise together with her fiancé, apart from yesterday where she felt she had to serve him as a reward for his being always so sweet to her. Had been feeling fine the first three days after she had been seeing me. Thursday had reported sick from school and is now at home again. This was very good because this way her fellow-students could see that she was physically ill with vomiting, so she has peace to heal her grief without having to account for them. We talk about that she is so perpetually self-extinguishing, devoted to duty and pleasing. She has to become an adult and make sure to know her own needs and get them fulfilled. On the plank bed we work at the time line in order to return to the original condition of being, pleasure and direction (zygote condition), and the patient manage to find this inner condition of tranquility and balance. The patient has now got this as a resource point. The patient suffers from /residual urine/ with several earlier cases of /pyelitis/. Has achieved orgasm only once with her fiancé; she is simply not so confident “south of the navel”. The uterus is “cold” and we work at it through the stomach.

7th conversation: Has chosen to postpone her imminent tiresome examination. She is very satisfied about this. We talk about that it is fine that she admit her desire. On the plank bed we work at the stomach and talk about what the stomach represents - existence, luck, desire and bliss. She still does not get so much advantage from sex. We talk about the G-point, and I ask her to explore herself and her sexuality. Mission/Exercise: We talk about love. I think of enneatype 9. Read “Maitri: The Spiritual Dimensions of the Enneagram”[43].

8th conversation: Has been emotionally very unbalanced with many problems on the home front (father) and does not feel to be able to manage her studies. I understand her difficulties, which I find temporary. On the plank bed we work at legs and back; discs in connection with slipped disc are being manipulated in their place.

9th conversation: Is very well for the time being after her crisis. However, she was very sad yesterday. Her shoulder hurts and is being relaxed, whereupon she cries. There are troubles with fiancé, who wants to have children with her and to move together, but she does not want. It seems as if she wants something else with the relationship than him and the question is whether they are suited to each other. On the plank bed we work at the stomach, which is obstreperous along colon decendens. I give: EXERCISE: When going to the lavatory, let go instead of pressing. May come back again in 14 days.

10th conversation: It went fine at her birthday. Nobody argued and everything was nice and quiet. She cried a little, because she wanted so badly to get love from her mother and father, but she did not get it. She has become much better in putting her father in his place. Dreamed she scolded him so much that he bled. Now he had truly got what he deserved. Her much older fiancé would so much like to have children of his own, narrowly she did prevent it. - He had already thrown her pills away, and then ... We talk about his motives. She has got no desire for him, but on the other hand for her former fiancé. Nevertheless she stays with the new fiancé because she depends on him. In my opinion it is a mess, and I say: “You must carefully look at what your needs are, be kind to yourself and fulfill your needs - in a completely selfish way. Stop thinking of him and his needs, think of your own”. OLD EXERCISES: It did not work well. She is not good enough with herself. She does not deserve it. “You are simply not worth it”, I say provokingly, and the tears squirt again. EXERCISE: Be good with yourself! If you want something - be sure to get it!

11th conversation: She arrives with a lot of power and determination today. We work on the plank bed at the stomach and breast regions. She contemplates to become a veterinary and we talk about this. Cries a little: I guess I cannot make it out. “Why not?” I ask. We also discuss her relationship and her fiancé writes: “I admire you because ... I love you because...” and we go into the issues and it seems that the patient would like to be loved. I see trouble ahead. EXERCISE: Say what you need. EXERCISE: Write more on your story. Childhood.

12th conversation: Has attended my courses *Philosophy of life that heals* II and III[10]. Has spontaneously relived many episodes of sexual abuse by her father at the age of 2-4 years, possible because of the fine support of a large group of participants. Has found her life purpose[19,35]: “I bring life and happiness”. She is doing very well and evolves exemplarily. Today we work at the stomach, which is tense. She has become massive and present, quiet and balanced. Her low back/slipped disc problem has on the whole disappeared now. A huge crack in the back today brought her vertebral column back in normal order.

13th conversation: Problems regarding her sexuality. The patient tells me it feels like a thousand knives around the vagina aperture. Gynaecologic examination: Nothing abnormal apart from 3 cm long scar corresponding to left labia minor at introitus [the

> vestibule]. At touching it, the patient goes into a dramatic gestalt with sexual abuse by her father - incest with full vaginal penetration - at the age of 4 years where she conceals herself in her hand. “I hide myself”. This sentence is let go by means of the roll. The scar is compatible with the incest episode taking place and the reliving of the episode is very intense. The patient has no longer low back/slipped-disc inconveniences, especially no pain. No nettle rash apart from a few elements occasionally. The uncontrollable episodes have disappeared. She feels like a healthy, natural and standard weighing young woman and does no longer weigh 3,000 tons. Her big problems connected with trust are now solved. The incest trauma was apparently the cause. Can be concluded if her condition is steady now.

This is one of the most difficult situations you may face as a holistic physician: when the patient goes directly into process (comes direct to expression) during the gynecologic examination when old scars in the sexual organs are touched physically. It is extremely important to have one’s own ethics and sexual borders in place as a holistic physician, otherwise one can easily get into trouble in situations like these. It is even better to have a safe procedure for handling this situation, which we therefore developed (called acupressure through the vagina, see below). When we have a suspicion that a patient has been exposed to sexual abuse, we adopt a particularly careful and methodical procedure that gives both the patient and the physician the safety required. The procedure involves training in backing out, visualizing the whole procedure in anticipation and support by an experienced nurse during the process.

> 14th conversation: Come with slight, fresh bleeding from rectum, which has lasted for a while. Rectal exploration: This examination provokes contact with the gestalt where father had anal coitus with the patient as she was about 2-3 years old. The feelings are very violent and mental projections very powerful with projections that I am “nasty” and “like them”. The assisting nurse at the clinic is called in and the contacted gestalt is dealt with together with her.

> 15th conversation: Home visitation in the evening together with the nurse after being called acutely, since the patient has experienced episodes of paranoid psychosis-like type where she did not dare to go out shopping because she was afraid - in mortal fear - to be assaulted and abused every time a man passed by. Did not at all dare to look at the shop assistant, who was a man, in the eyes or touch his hands. Was horror-struck to be sent to psychiatric department. Timeline therapy is adopted and I work at the scar in the mouth which derives from the patient having bitten herself during the infringements - there are distinct 1 cm big scars in each cheek corresponding to reiterated bites, and one very hard 5 mm big scar in the lower lip just at the left from the middle corresponding to a bite. Timeline therapy is processed with about 100 accomplished intercourses with father and two other men mentioned below. Patient says “I don’t want to” “I can no more” “I don’t want any more”. About five accomplished intercourses with uncle as she was 2-5 years old, she tells. “He is so disgusting” “He is so cold”. He says “So, here you are, now you shall have it” - this made the patient very furious as she did not want it - and “This is reckless” “I cannot feel it”. And a little later – “Then he says: Now, nobody will want to have you”. She is clearly awfully afraid they will leave her alone now. It was most traumatic when her uncle took her alternately in anus and vagina in a very quick assault where the patient could not at all manage to find her bearings, carried out with huge violence. She explains: “He takes me alternately in one hole and the other. I cannot stand on my feet after that. I’m afraid to get ill as one may only dry oneself one way. I cannot touch it. - It is as if, if I touch, it bursts. The whole is going to pieces.” He took her three times in the bathroom and twice earlier in the shop. About five accomplished intercourses with grandfather in the basement, she tells: He says: “This is a "lamb thigh”. “It is not a lamb thigh." - and later the patient says: He says afterwards: "I will kill you if you tell anybody." The patient is emotionally affected, with massive shame and guilt. Strongly agitated for many hours. Afterwards very relieved. We are processing the whole time track hour by hour and about 100 incest infringements are exposed, with coitus at the age of 2-5 years with the three above-mentioned perpetrators (father, grandfather and uncle). It seems as if the three men have shared the patient sexually, with her father's knowledge and approval, but this is my interpretation based on details from the progress of events: The father comes with the patient to his elder brother, who is allowed to take her to the back of the shop alone where the infringements took place. The father comes with the patient to his own father who is let to take her to the basement alone where the infringements are performed.

16th conversation: I work at the scar in the mouth, which arouse new memories of infringements.

17th conversation: Fit of paranoia again; I suspect that she gets bad in order to insist on care. Has to see a psychiatrist if paranoia continues.

18th conversation: "I have not felt like this before. I have always been uncertain. Have always let others control me. Now it is totally different and now only I myself am at the helm. And then, I am happy." Is evaluated to be through now. May come and see me again if required.

Both during the therapy and also afterwards, contact has been maintained via the phone. She called several times during the months after termination of treatment to discuss thoughts and feelings before she felt confident enough to let go of the therapeutic connection definitively. In a following paper, the patient's own description via her diary will be presented.

## DISCUSSION

The most amazing observation from this experience is that healing of even the most terrible of traumas does not take a long time if the patient enters the very special state of consciousness we call holistic healing. Years of the most terrible abuse can be healed in only hours of such intensive therapy. The problem is to get the patient into the process because this takes a lot of trust, which this particular patient did not have, since sexual abuse is the exact situation where a person in power (a parent, employer, adult) abuses the trust and crosses the line called interhuman trust. Winning the patient's trust is really the art of holistic medicine and this will only happen if you love your patients, very much in the same way as you love your children (love is for some a very strong word, but we think it is the right word for the intense care that a good physician must give to his patients in order to be able to help them).

Another important lesson to learn seems to be the fact that many abused girls have completely repressed the episodes of abuse. The repression of trauma containing so much emotional charge can literally cause insanity. So when a young girl shows the signs of nervous breakdown or a borderline picture or symptoms, it is wise to take the possibility of early and repressed incest or other sexual abuse into consideration. A third important observation is that the process of healing needs an extreme amount of trust and resources. Only in a safe and loving environment can the patient heal. A fourth important thing to notice is that a psychotic episode in the middle of therapy can be taken as old emotional pains reappearing and thus a sign that the patient is healing. It is important that this natural and spontaneous process of regression is not blocked by antipsychotic drugs, but that both physicians and the nurses of the team understand that this is a sound and natural reaction and yet another invitation to give intensive holding. Insanity in the form of acute psychoses in the course of holistic existential therapy is a sign of intensive healing.

If such infinite closeness and intimacy (or love as we call it) is possible, then the precondition for successful holding can be established in all the five dimensions: care, respect, awareness, acceptance, and acknowledgment. The physical closeness is necessary for the healing because without contact, there can be no giving or receiving of physical and sexual acceptance, which is what the incest or sexual abuse victim needs more that anything.

The problems of ethics are obvious and severe. All the procedures involving contact with genitals must be carried out in standardized and safe ways[9,27,28,44]. As the patient with very severe repressed sexual traumas is likely to project the content of the old gestalt at the same moment that the physician touches the involved area, the patient will perceive the physician/therapist and his helpers as abusers in the present moment — often at the same time knowing that this is not true, but in fact only a projection, as in Anna's case. The therapist must not use a defensive attitude at any time, but must be regretful and open to all the criticism that is presented by the patient at that moment. Only by taking full responsibility can the therapist help the patient through the most painful of the traumas. Assuming responsibility as a holistic therapist for causing the patient to re-experience earlier incidents of rape or sexual abuse, even when projected into present time, is called "controlled sexual abuse" in the advanced toolbox for holistic medicine (a advanced level 8 technique[9], which is a variation of the technique of acupressure though the vagina[44]). To use this high-level technique takes a lot of experience and courage. We strongly recommend intensive training in the 10 levels of holistic medical tools[9] and supervision of the therapist during such work. It is also very important to follow the laws and regulations of the country in which you work. In some countries, holistic gynecological procedures as described above might be illegal to carry out in general medical practice.

## CONCLUSION

The nervous breakdown of a young woman was caused by the spontaneous integration of repressed, severe sexual abuse in her childhood. As the repressed material manifested itself, she became more and more emotionally labile and extreme vulnerable. The tools of the advanced toolbox for holistic medicine were successfully taken into use. During the holistic existential therapy, the patient accumulated resources to integrate the old trauma. After the recovery of her human character and purpose of life in accordance with the life mission theory, the theory of human character, and the holistic process theory of healing, she finally was able to confront and integrate her old emotional pains and heal the wounds of her existence. The case story of Anna is an example of how even the most severely ill patient can recover fully with the support of holistic medical treatment, making her feel, understand, and let go of her negative beliefs and life-denying decisions.

## ACKNOWLEDGMENTS

This study was supported by grants from IMK Almene Fond. The quality of life research was approved by the Copenhagen Scientific Ethical Committee under number (KF)V.100.2123/91.

## BIOSKETCHES

**Søren Ventegodt, MD,** is the director of the Quality of Life Research Center in Copenhagen, Denmark. He is also responsible for a Research Clinic for Holistic Medicine in Copenhagen, is a popular speaker throughout Scandinavia, and is a teacher at the Nordic School of Holistic Medicine. He has published numerous scientific or popular articles and a number of books on holistic medicine, quality of life, and quality of working life. His most important scientific contributions are the comprehensive SEQOL questionnaire, the very short QoL5 questionnaire, the integrated QOL theory, the holistic process theory, the life mission theory, the theory of human character, and the Danish Quality of Life Research Survey, 1991–94 in cooperation with the University Hospital of Copenhagen and the late professor of pediatrics, Bengt Zachau-Christiansen, MD, PhD. E-mail: ventegodt@livskvalitet.org. Website: http://www.livskvalitet.org

**Birgitte Clausen, RN,** is leader of a secluded ward for people with dementia, teacher at the Nordic School of Holistic Medicine, and dementia coordinator and consultant in Aarhus, Denmark with more than 20 years of experience in adult psychiatry and dementia. During the last 4 years, she worked together with Søren Ventegodt on the "QOL as medicine project" and on developing existential holistic group therapy. Through her articles and lectures, she has disseminated "The New Culture of Dementia" in both

Denmark and Japan. She has participated in the production of several videos to order to inspire relatives, medical staff, and volunteers to understand people with dementia. This project has been granted financial support from the Ministry of Social Affairs in Denmark. She has conducted the first Danish investigation on the effect of sufficient daylight on the human spirit, activity level, and biological rhythm. E-mail: villahvide@mail.dk

**Joav Merrick, MD, DMSc,** is professor of child health and human development affiliated with the Center for Multidisciplinary Research in Aging, Zusman Child Development Center, Division of Pediatrics and Community Health at the Ben Gurion University, Beer-Sheva, Israel; the medical director of the Division for Mental Retardation, Ministry of Social Affairs, Jerusalem; and founder and director of the National Institute of Child Health and Human Development. He has numerous publications in the field of child health and human development, rehabilitation, intellectual disability, disability, health, welfare, abuse, advocacy, quality of life and prevention. Dr. Merrick received the Peter Sabroe Child Award for outstanding work on behalf of Danish Children in 1985 and the International LEGO-Prize ("The Children's Nobel Prize") for an extraordinary contribution towards improvement in child welfare and well being in 1987. E-mail: jmerrick@internet-zahav.net. Website: www.nichd-israel.com
